# Self-regulated learning, online mathematics learning engagement, and perceived academic control among Chinese junior high school students during the COVID-19 pandemic: A latent profile analysis and mediation analysis

**DOI:** 10.3389/fpsyg.2022.1042843

**Published:** 2022-11-03

**Authors:** Wenwu Dai, Zhaolan Li, Ning Jia

**Affiliations:** College of Education, Hebei Normal University, Shijiazhuang, China

**Keywords:** self-regulated learning, online mathematics learning engagement, perceived academic control, COVID-19, latent profile analysis

## Abstract

**Objectives:**

Under the COVID-19 prevention and control policy, online learning has been widely used. The current study aimed to identify latent profiles of self-regulated learning in the context of online mathematics learning during the recurrent outbreak of COVID-19, and examine the mechanisms underlying the relationship between self-regulated learning and online mathematics learning engagement among Chinese junior high school students using variable-and person-centered approaches.

**Methods:**

A sample of 428 Chinese junior high school students (47.66% female) completed questionnaires on self-regulated learning, perceived academic control, and learning engagement. Mplus7.0 was used to analyze the latent classes of self-regulated learning. A mediation model was conducted using the software SPSS PROCESS macro.

**Results:**

Three profiles of self-regulated learning were identified and named as low self-regulated learning (16.12%), medium self-regulated learning (43.23%), and high self-regulated learning (40.65%). In the mediating analysis, results of the variable-centered approach showed that perceived academic control mediated the effects of self-regulated learning on learning engagement. For the person-centered approach, we selected the low self-regulated learning type as the reference profile, and the analysis revealed that compared with the reference profile, perceived academic control partially mediated the link between the medium self-regulated learning profile and learning engagement; perceived academic control partially mediated the relationship between the high self-regulated learning profile and learning engagement.

**Conclusion:**

This study showed the heterogeneity in the online mathematics self-regulated learning patterns of Chinese junior high school students during the COVID-19 pandemic, revealing the internal mechanisms of Chinese junior high school students’ online mathematics learning engagement using variable-and person-centered approaches. Furthermore, the findings of the study have important implications for promoting online mathematics learning engagement among junior high students during the pandemic.

## Introduction

The COVID-19 outbreak has disrupted traditional classroom teaching (Wijaya et al., 2020). Since the introduction of the Chinese Ministry of Education’s “Home Study” Initiative in mid-February 2020,^1^ the development of online education has been accelerated. Online learning is a key component of the advancement of education and teaching through information technology. Online learning provides learners with more convenience and autonomy (Mullen, 2020), but the quality of online learning is often inferior to traditional, face-to-face instruction (Spencer and Temple, 2021). Online learning engagement is a significant metric for determining the quality of online education (Moubayed et al., 2020). However, enhancing students’ engagement in online learning and improving the efficiency of online learning has become a difficult problem in teaching management during the epidemic (Park and Yun, 2018). Due to the specificity of learning engagement (Martin, 2008), most previous studies have ignored its characteristics and only investigated learning engagement in general learning situations (Jiang et al., 2018). Mathematics is a fundamental subject of basic education that is highly abstract and relies on stringent logic. Learning mathematics is often one of the most difficult subjects for junior high school students (Stodolsky et al., 1991). It can be quite challenging for all students. Online mathematics learning engagement can not only predict the mathematics achievement of online learners (Phan et al., 2016), but also promote the subsequent integration of offline mathematics classes (Pellas, 2014). And mathematics learning engagement is a sub-field of learning engagement (Greene, 2015), the current situation and influence mechanism of junior high school students’ online mathematics learning engagement is becoming one of the hot issues that online educators pay attention to.

The motivation and engagement wheel model and empirical studies support that self-regulated learning plays an important role in online mathematics learning engagement (Martin, 2007). Most previous studies on self-regulated learning have taken a variable-centered perspective, which does not account for potential variability and differences in variables between individual. Hence, in order to better explore the heterogeneity of self-regulated learning, the current study focused on the context of online mathematics learning and adopted a variable-centered approach to explore the latent classes of self-regulated learning among junior high school students. We also examined whether significant differences existed among profiles with regard to online mathematics learning engagement. There are a few studies into the mediating mechanism of self-regulated learning on online mathematics learning. Based on the social cognitive theory and the motivation and engagement wheel model, this study explored the mediating role of perceived academic control in the relationship between self-regulated learning and online mathematics learning. Using variable-and person-centered approaches, we have found that self-regulated learning is divided into three latent classes, and perceived academic control plays a mediating role between self-regulated learning and online mathematics learning. Our study offered educators and researchers valuable insights into understanding distinctive patterns of junior high students’ self-regulated learning and the impact mechanism of online mathematics learning engagement.

## Literature review

### Mathematics online learning engagement and self-regulated learning

Mathematics online learning engagement refers to students’ active participation in mathematical learning activities, deep thought, energetic coping with challenges and setbacks, and positive emotional experiences during the learning process (Fredricks et al., 2004; Liu et al., 2018), which mainly involves three dimensions: behavioral engagement, emotional engagement, and cognitive engagement. Among them, behavioral engagement describes the behavior of students involved in mathematics learning activities, such as participation, concentration, and completing motivation and engagement wheelwork on time; cognitive engagement refers to the psychological involvement of students and the cognitive strategies employed to grasp complex concepts; and emotional engagement refers to students’ positive emotional responses to teachers, classmates, and online learning activities. The research on the influencing factors of mathematics learning engagement is still in its early stages (Greene, 2015). In a qualitative study of teenagers’ online learning engagement, it is found that few students can effectively participate in online learning, and most students encounter problems in online learning engagement (Li et al., 2021). Therefore, it is necessary to explore the influencing factors and psychological mechanisms of junior high school students’ online mathematics learning engagement, which has practical significance for improving their mathematics learning engagement.

Recently, researchers have demonstrated that many learner characteristics and external factors can effectively predict student online learning engagement. The factors included the quality of teacher-student interaction (Lu and Churchill, 2014), peer relationships (Shao and Kang, 2022), self-handicapping (Holliman et al., 2018), and self-regulation (Stan et al., 2022), among others. Among the preconditions that influence online mathematics learning engagement, self-regulating learning (SRL) is especially important. Self-regulated learning refers to the process in which learners constantly stimulate learning motivation, improve learning initiative, and use appropriate and effective strategies for learning (Zimmerman, 1989; Wijaya et al., 2020). Typical self-regulated learners often use cognitive and metacognitive strategies to achieve their learning goals; they efficiently manage time and study environment to optimize their academic performance; and they proactively seek support from teachers and classmates when they encounter difficulty (Pintrich and De Groot, 1990; You and Kang, 2014). Numerous theoretical and empirical studies have linked self-regulated learning to online mathematics learning engagement. The impact of self-regulated learning on Engagement in online mathematics learning can be explained by the motivation and engagement wheel model (Martin, 2007). The model was divided into two parts: motivation and engagement, including adaptive behavior, maladaptive behavior, adaptive cognition, and maladaptive cognition. According to Martin, motivation belongs to adaptive cognition, which has a positive impact on the adaptive behavior of learning engagement. A study on learning engagement in online mathematics courses found that learning motivation can promote students’ learning engagement through self-management and control of effort and attention, and adjustment of learning strategies (Kim et al., 2015). Similarly, an empirical study has also found that self-regulated learning has a significant impact on learning engagement (Stan et al., 2022). Based on the above literature, we propose our first hypothesis: *There is a significantly positive correlation between self-regulated learning and online mathematics learning engagement among junior high school students.*

### Investigation of the profiles of self-regulated learning

To date, most studies investigating the relationship between self-regulated learning and learning engagement in online learning environments have employed a variable-centered approach, which does not account for potential variability and differences in variables between individuals (Wang et al., 2021). Instead, as a person-centered analysis technique, the latent profile analysis (LPA) groups individuals into latent classes or profiles or subgroups according to the correlations on self-regulated learning (Luo et al., 2021). This research method helps to provide a clear understanding of the self-regulation learning of junior middle school students in online mathematics classes, which guides intervention work based on the unique needs of each group instead of a single target variable. Previous studies have preliminarily explored the latent classes of self-regulated learning. Using student motivational, regulatory, and contextual variables, the cluster analysis method was adopted to explore different combinations of motivation/self-regulation variables and contextual variables (Cleary et al., 2021). The study found that students mainly were divided into four classes in mathematics learning activities: High SRL-high Support, Solid SRL-low Support, Very Low SRL-low Support, and Low SRL-supported. However, their study mainly explored different combinations of motivation/self-regulation variables and contextual variables but did not explore the latent classes of self-regulated learning in students’ learning activities.

Recently, a study based on the context of a blended learning environment used LPA to analyze the latent classes of self-regulated learning among online learners and found that there are three latent classes of self-regulated learning among online learners: high, low, and moderate self-regulated learning profiles (Vanslambrouck et al., 2019). Blended learning environments combine face-to-face and online learning activities to complement each other (Hrastinski, 2019), suggesting that the latent classes of online self-regulated learning may be analogous to those in blended learning environments. Therefore, we propose our second hypothesis: *There are three latent classes of junior high school students’ online mathematics self-regulation learning, which are high, medium, and low self-regulation learning.* After identifying the latent classes of self-regulated learning, we compared the differences between different profiles in the perceived academic control and learning engagement in online mathematics learning among junior high school students.

### The mediation of perceived academic control

There may be a mediating mechanism between self-regulated learning and online mathematics learning engagement. Perceived academic control is a vital variable. Perceived academic control is an individual’s subjective assessment of their ability to control and predict academic outcomes (Perry et al., 2001). It is a branch of academic self-efficacy (Wang et al., 2017). The motivation and engagement wheel model shows that academic self-efficacy belongs to adaptive cognition (Martin, 2007), and it can also have a positive impact on learning engagement. This indicates that high perceived academic control provides individuals with a stable psychological learning environment and increases their sense of security, which is conducive to improving their confidence in learning, helping individuals to dig their potential more intently, and promoting high-quality learning (Jiang et al., 2018). Similarly, perceived academic control reflects a sense of control over academic outcomes. When people lose a sense of control over the results of their behavior, they are likely to reduce their expectations for the future and generate tired emotional experiences, thus hindering positive learning engagement. The findings of an empirical study showed that primary school students’ perceived academic control not only directly predicted the degree of mathematical learning engagement, but also had an impact on mathematical learning engagement through expectation (Jiang et al., 2018). Moreover, the results of a study found that perceived internal locus of control in an online learning environment significantly predicted learning persistence (Joo et al., 2011).

On the other hand, the motivation and engagement wheel model does not specify the relationship between motivation and efficacy, but the relationship between self-regulated learning and perceived academic control can be explained by the social cognitive theory (Bandura, 1986). The model suggests that self-regulation and self-efficacy are both keys to engagement, and the behaviors related to self-regulation learning, such as task selection and learning strategies, will affect self-efficacy. This indicates that in the process of self-regulated learning, students show a reasonable plan for learning progress and effective use of strategies, which is conducive to promoting perceived academic control. This relationship has been tested in the sample based on middle school students (Jiang et al., 2020). Based on the above findings, we propose our third hypothesis: *Perceived academic control plays a mediating role between self-regulated learning and online mathematics learning engagement among junior middle school students.* And we will verify the mediating effect of the above hypothesis based on two research methods—variable-centered and individual-centered.

## The current study

Combining the motivation and engagement wheel model and the social cognitive theory, the present study sought to explore the online mathematics learning experiences of junior high school students, using variable-centered and individual-centered approaches to data collection and analysis. We identify potential patterns of self-regulation that could influence online learning engagement and explore whether perceived academic control mediates the relationship between self-regulated learning and online learning engagement.

## Materials and methods

### Participants and procedures

The sample was obtained through random cluster sampling, 454 junior high school students with online mathematics learning experience were conducted on the online survey platform (Wen Juan Xing) from three junior high schools in Hebei Province. The informed consent of the person in charge of the school was obtained before the test was administered, and an anonymous survey was used to ask the subjects to answer truthfully and independently. According to the two criteria of a consistent reaction and a response time outside of the plus or minus 3 standard deviation range, we deleted the waste questionnaires. 428 valid questionnaires were obtained, with an effective recovery rate of 94.27%, including 224 males (52.34%) and 204 females (47.66%).There were 235 (54.91%) seventh grade students and 193 (45.09%) eighth grade students. Informed consent was obtained from all participants and no compensation was provided to participants. This study was approved by the Ethics Committee of Hebei Normal University. All participation in this study was voluntary, and informed consent was obtained from parents in advance. Table 1 shows the complete data on the participants.

**Table 1:** Descriptive statistics of junior high school student respondents.

Items	Type	*N*	Percentage
Sex	Male	224	52.34%
	Female	204	47.66%
Grade	7	235	54.91%
	8	193	45.09%
Total		428	100%

### Measurements

#### Self-regulated learning

The self-regulated learning Questionnaire (SRL) was developed by Barnard et al. (2009), and the Chinese version has been proved to be reliable (Fung et al., 2018). It has 24 items and is a five-point-Likert scale (1 = “strongly disagree,” 5 = “strongly agree”). According to the research purpose and the actual situation of online mathematics learning, this study deleted two questions in the original questionnaire: “I set standards for my assignments in online courses” (Goal setting dimension) and “Although we do not have to attend daily classes, I still try to distribute my studying time evenly across days” (Time management dimension). This revised questionnaire included six subscales: Goal setting (Questions 1-4), environment structuring (Questions 5–8), task strategies (Questions 9–12), time management (Questions 13–14), help-seeking (Questions 15–18), and self-evaluation (Questions 19–22). The Cronbach’s alpha for OSRL was 0.920. For confirmatory factor analysis of this scale, the fit index was as follows: *x*^2^/*df* = 4.823, RMSEA = 0.085，NFI = 0.835，IFI = 0.864, TLI = 0.837, and CFI = 0.863. The above indicators showed that the reliability and validity of the revised scale were acceptable and basically meet the requirements of psychometrics.

#### Online mathematical learning engagement

A 13-item Mathematical Learning Engagement Scale (MLES) was applied to assess the degree of online mathematics learning engagement (Chai and Gong, 2015). It had three dimensions (cognitive engagement, emotional engagement, and behavioral engagement) rated on a five-point-Likert scale (1 = “strongly disagree,” 5 = “strongly agree”), including two reverse scoring items. According to the actual situation of online mathematics learning, we have added a description of online to each item. The Cronbach’s alpha for MLES was 0.855.

#### Perceived academic control

An eight-item Perceived Academic Control Scale (PACS) was developed by Perry et al. (2001), and the Chinese version has been proved to be reliable (Ju, 2012). It had two dimensions (external control and internal control) rated on a five-point-Likert scale (1 = “strongly disagree,” 5 = “strongly agree”). According to the actual situation of online mathematics learning, we have added a description of online mathematics to each item. The Cronbach’s alpha for PACS was 0.800.

### Statistical analysis

All data were analyzed using SPSS25.0, AMOS24.0, and Mplus7.0. First, descriptive statistics and Pearson correlation analysis were performed on all variables. Second, we performed an LPA analysis based on the item level, identifying self-regulated learning of different clustering patterns; when using Mplus7.0 for LPA, we evaluated one-to five-class groups and compared them based on the fitting index. A good class should have the following conditions: (1) lower Akaike Information Criterion (AIC) values, lower Bayesian Information Criterion (BIC) values, and lower sample size-adjusted BIC values (aBIC); (2) significant Lo–Mendell–Rubin likelihood test (LMR) *p* values, significant adjusted Bootstrapped Likelihood Ratio Test (BLRT) *p* values; and (3) higher entropy values with numbers closer to 1; Third, after determining the best class by LPA, using univariate ANOVAs and *post-hoc* tests to compare differences in perceived academic control, and online mathematics learning engagement among the self-regulated learning profiles. Fourth, a simple mediation analysis of perceived academic control mediating the relationship between self-regulated learning and online mathematics learning engagement was tested using Hayes’s PROCESS 3.2 macro for SPSS (Model 4).

To reduce common method bias due to participant self-report, this study controlled for it procedurally and statistically. In terms of procedures, this study used anonymous surveys and reverse scoring of some items to carry out certain controls; in terms of statistics, we examined common method variance. Harman’s single factor test found that the first variance explanation rate was 35.57%, which is less than the critical value of 40%, indicating no significant common method bias in this study (Zhou and Long, 2004).

## Results

### Preliminary analysis

First, referring to previous research practices (Wijaya et al., 2022b), we performed descriptive statistics on the measured variables in order to respond to the characteristics of participants’ answers. The mean, standard deviation, skewness, and kurtosis are included in the descriptive statistics results (see Supplementary Table 1, for more details). According to the previous study, the data considered being normal for the range of skewness from −2 to +2 and the range of kurtosis from −7 to +7 (Byrne, 2010; Hair et al., 2010). Skewness and kurtosis in this study were within the acceptable value range.

Second, we analyzed the correlation of the three variables. Table 2 showed the descriptive statistics and correlation matrix for the study variables. The results showed that self-regulated learning was positively associated with perceived academic control and online mathematics learning engagement (*p <* 0.001). perceived academic control was positively associated with online mathematics learning engagement (*p <* 0.001). The correlation coefficients between any two of the three variables were lower than 0.8, which indicates that low-to-moderate correlation existed between the variables, and no collinearity was observed (Benesty et al., 2009).

**Table 2:** Means (M), standard deviations (SD), and correlations (*N* = 428) among the major variables in this study.

	*M*	*SD*	1	2	3	4
1.Gender	—	—	1	—	—	—
2.Self-regulated learning	3.815	0.673	−0.047	1	—	—
3.Perceived academic control	3.533	0.691	−0.149^**^	0.652^***^	1	
4.Online mathematical learning engagement	3.877	0.675	−0.055	0.753^***^	0.606^***^	1

^**^p < 0.01 and ^***^p < 0.001.

### LPA results

**Figure 1:**
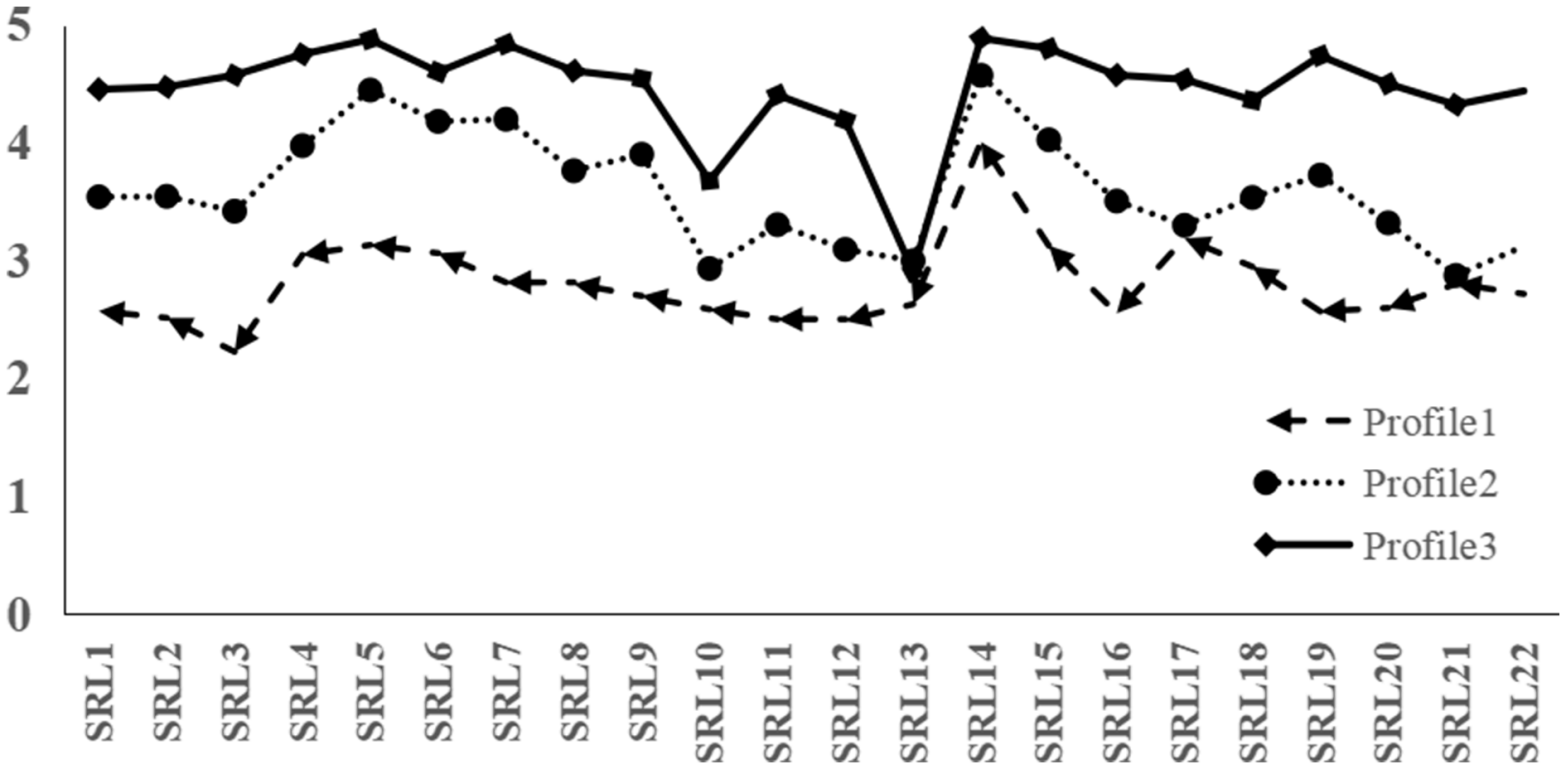
The three profiles of self-regulated learning by latent profile analysis.

The fitting indices of the five LPA models are showed in Table 3. The three-profile model had lower AIC, BIC, and aBIC values than the two-profile model and had significant values of *p* for BLRT and LMR. Although the four-profile model had lower AIC, BIC, and aBIC values than the three-profile model and had significant values of *p* for BLRT, the downtrend of AIC, BIC, and aBIC became slow, the entropy was less than that of the three profile model. In addition, according to the parsimonious guideline, the three-profile solution was chosen as the final model. According to the characteristics of the model (Figure 1), the subgroups were named as low self-regulated learning (Profile 1,16.12%), medium self-regulated learning (Profile 2, 43.23%), and high self-regulated learning (Profile 3, 40.65%).

**Table 3:** Fitting index and group size of latent analysis models.

Indices	Unconditional model				
	1-Class	2-Class	3-Class	4-Class	5-Class
Fit statistics					
AIC	28330.863	25873.139	**25134.285**	24776.544	24473.339
BIC	28509.465	26145.100	**25499.606**	25235.225	25025.379
aBIC	283369.836	25932.483	**25214.000**	24876.632	24593.798
Entropy	—	0.925	**0.938**	0.934	0.921
BLRT (*p*)	—	<0.001	**<0.001**	<0.001	<0.001
LMR (*p*)	—	<0.001	**0.015**	0.406	0.774
Group size(%)					
C1	428(100.00%)	196(45.79%)	**69(16.12%)**	87(20.33%)	20(4.67%)
C2	—	232(54.21%)	**185(43.23%)**	16(3.74%)	91(21.26%)
C3	—	—	**174(40.65%)**	170(39.72%)	142(33.18%)
C4	—	—	—	155(36.21%)	36(8.41%)
C5	—	—	—	—	139(32.48%)

Indices of the best-fitting model are in boldface.

### Profile differences in perceived academic control and online mathematics learning engagement

The differences in perceived academic control and online mathematics learning engagement among the three profiles were examined by using ANOVAs. The high self-regulated learning profile had the highest perceived academic control. The medium self-regulated learning profile had middle-level perceived academic control. The low self-regulated learning profile had the lowest perceived academic control. The profiles also differed overall in terms of online mathematics learning engagement. The high self-regulated learning profile had the highest online mathematics learning engagement. The medium self-regulated learning profile had middle-level online mathematics learning engagement. The low self-regulated learning profile had the least amount of online mathematics learning engagement (Table 4).

**Table 4:** Differences in Perceived academic control and online mathematics learning engagement across latent profiles (M ± SD).

	Profile1 (*N* = 69)	Profile2 (*N* = 185)	Profile3 (*N* = 174)	*F*	*η* ^2^	*Post hoc*
Perceived academic control	2.734 ± 0.672	3.408 ± 0.544	3.984 ± 0.691	144.092	0.404	1 < 2 < 3
Online mathematics learning engagement	2.895 ± 0.572	3.806 ± 0.399	4.341 ± 0.469	248.819	0.539	1 < 2 < 3

### Mediated effects of perceived academic control

Before model fitting, we performed a Z-score transformation on the three variables. The PROCESS3.2 macro in SPSS25.0 was used to analyze the mediation effect of perceived academic control (Hayes, 2018). Simple mediation analysis was used to analyze mediating analysis based on variable-centered, and multi-categorical mediation analysis was used to analyze mediating analysis based on person-centered.

### Mediation analyses: Variable-centered approach

**Figure 2:**
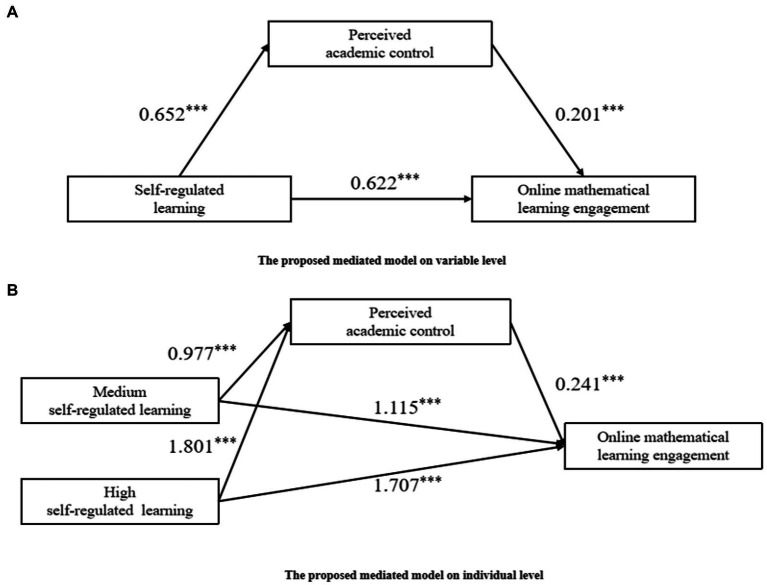
**(A)** The proposed mediated model on variable level. **(B)** The proposed mediated model on individual level. ***p < 0.001.

Figure 2A showed the results of this test. Self-regulated learning significantly and positively predicted online mathematics learning engagement (*β =* 0.652, *p <* 0.001); When self-regulated learning and perceived academic control entered the equation at the same time to predict online mathematics learning engagement, both self-regulated learning and perceived academic control significantly and positively predicted online mathematics learning engagement (*β =* 0.622, *p <* 0.001; *β =* 0.201, *p <* 0.001). These results suggest that perceived academic control partially mediates the effect of self-regulated learning on online mathematics learning engagement.

Bias-corrected non-parametric percentile bootstrapping was conducted to test the mediating effect of perceived academic control. An indirect effect value of 0.131 and a 95% CI of 0.085–0.179. The mediating effect accounted for 17.41% of the total effect, which indicates the significance of the mediating effect of perceived academic control.

### Mediation analyses: Person-centered approach

Two dummy variables (medium self-regulated learning and high self-regulated learning) were created by using indicator coding to represent the three self-regulated learning profiles. The low self-regulated learning profile served as a reference group. The omnibus mediation effects [*F* (2, 425) = 248.819, *p* < 0.001] and the relative mediation effect [*F* (2, 424) = 102.521, *p* < 0.001] were significantly different from zero.

The 95% bootstrap CI (0.136, 0.346) of the relative mediation of the relationship between medium self-regulated learning and on online mathematics learning engagement excluded 0, which indicated that the relative mediation effect was significant (a_1_ = 0.977, *p* < 0.001; *b* = 0.241, *p* < 0.001; a_1_b = 0.235; Figure 2B). That is, junior high school students with medium self-regulated learning had 0.977 times greater perceived academic control than those with low self-regulated learning (a_1_ = 0.977), and their online mathematics learning engagement level also increased by 0.241 (b = 0.241) with perceived academic control. The relative direct effect of medium self-regulated learning was significant (c’_1_ = 1.115, *p* < 0.001), which indicated that online mathematics learning engagement in students with medium self-regulated learning was 1.115 higher than that of students with low self-regulated learning. Similarly, the 95% bootstrap CI (0.275, 0.603) of the relative mediation of the relationship between high self-regulated learning and online mathematics learning engagement excluded 0, which indicated that the relative mediation effect was significant (a_2_ = 1.801, *p* < 0.001; b = 0.241, *p* < 0.001; a_2_b = 0.435). That is, junior high school students with high self-regulated learning had 1.801 times greater perceived academic control than those with low self-regulated learning (a_2_ = 1.801), and their online mathematics learning engagement level also increased by 0.241 (b = 0.241) with perceived academic control. The relative direct effect of medium self-regulated learning was significant (c’_1_ = 1.707, *p* < 0.001), which indicated that online mathematics learning engagement in students with high self-regulated learning was 1.707 higher than that of students with low self-regulated learning.

## Discussion

Most previous studies on self-regulated learning have taken a variable-centered perspective, which does not account for potential variability and differences in variables between individual. Hence, in order to better explore the heterogeneity of self-regulated learning, the current study focused on the context of online mathematics learning and adopted a variable-centered approach to explore the latent classes of self-regulated learning among junior high school students. Although motivation and engagement wheel model and a large number of empirical studies support that self-regulated learning plays an important role in online mathematics learning engagement, there are a few studies into the mediating mechanism of self-regulated learning on online mathematics learning. Based on the social cognitive theory and the motivation and engagement wheel model, this study explored the mediating role of perceived academic control in the relationship between self-regulated learning and online mathematics learning. Using variable-and person-centered approaches, we have found that self-regulated learning is divided into three latent classes, and perceived academic control plays a mediating role between self-regulated learning and online mathematics learning.

### Self-regulated learning profiles

We found the latent classes of self-regulated learning by conducting an LPA. Consistent with our hypothesis, the LPA revealed three profiles of self-regulated learning among junior high school students: low self-regulated learning (16.12%), medium self-regulated learning (43.23%), and high self-regulated learning (40.65%). These profiles differed from one another in terms of perceived academic control and learning engagement. Based on the context of online mathematics learning, this study found that the latent classes of self-regulated learning among junior high school students were partially consistent with the results of previous studies. The number of latent classes of self-regulated learning is consistent with the results of previous studies, but the trend in some dimensions is different from that of previous studies (Vanslambrouck et al., 2019). According to Figure 1, the scores of the three self-regulated learning profiles in the time management dimension were comparable, which was inconsistent with the previous research results. Vanslambrouck et al. (2019) found that low and moderated self-regulated learning profiles used fewer time strategies compared to the overall mean self-regulated learning. The main reasons for this difference were as follows: the previous research was mainly based on a blended learning environment, the learning environment allowed students to have greater autonomy, and students could arrange independently their own schedules. But our study was based on the background of junior high school students’ online learning in which teachers have arranged for online learners’ study plans and schedules (Munastiwi and Puryono, 2021), resulting in similar time management among the three profiles of self-regulated learning. This result was also in accordance with the deletion of relevant questions from the self-regulation questionnaire. It was further found that low and medium self-regulated learning profiles scored similarly on items 17 and 21. However, we could not reach a definitive conclusion as to whether this was consistent with previous studies. Because we conducted LPA on items, rather than dimensions as in previous studies. Further research is needed to analyze the difference.

### Relationship between self-regulated learning and online mathematics learning engagement

The current study found a significant positive correlation between self-regulated learning and engagement in online mathematics learning among junior high school students. This is consistent with the motivation and engagement wheel model, which suggests that these two factors are linked. The Chinese mathematics curriculum reform emphasizes the importance of student-centered learning activities, which highlights the importance of student initiative in mathematics learning. And compared to traditional classroom learning, online learning is more student-centered and requires students to manage their own learning (Barnard et al., 2009). Learners who are good at self-regulation can accurately analyze learning tasks, clarify learning objectives, search for relevant information, and evaluate the learning process according to learning objectives, thus effectively increasing the degree of learning engagement.

### Mediation of perceived academic control

Based on a variable-centered approach, this study also found that perceived academic control mediates the relationship between self-regulated learning and online mathematics learning engagement, confirming our proposed hypothesis. Self-regulation is a cyclic process, sufficient prior preparation provides the individual with mathematical learning goals and motivation, urges the individual to focus on solving mathematical problems, self-evaluates the target results obtained by himself, and thus affects the subsequent preparation of the individual (Jiang et al., 2020). In this process, online learners with a high level of self-regulation can acquire a stronger sense of goal, and better complete online mathematics learning tasks, which are conducive to improving the individual’s perceived academic control. Faced with the increased difficulty and number of knowledge points in mathematics, junior high school students tend to have negative beliefs about their ability to learn the subject. When an individual has a high sense of control in online mathematics learning, such a high perceived academic control helps the individual to cultivate positive beliefs, which plays an important role in students’ mathematics learning difficulties. Moreover, individuals with high perceived academic control in their studies will promote them to realize their responsibilities in online learning (You and Kang, 2014). The combination of the two makes individuals invest more cognition, emotion and behavior in the learning process; On the contrary, when individuals lose their sense of control, they will lose their interest and confidence in learning mathematics and feel bored (Putwain et al., 2013), and this weariness will limit their attention and damage their cognitive resources. Compared with the learning of other subjects, the learning of mathematics needs more abundant cognitive resources (Putwain et al., 2013). Therefore, when individuals lose their sense of control, it is not conducive to the in-depth processing of mathematical knowledge points, and further hinders students’ investment in mathematical learning.

Another important finding of this study is that the effect of differences between high/medium and low self-regulated learning profiles on online mathematics learning engagement is partly mediated by perceived academic control. Compared with low self-regulated learning profile, high and medium self-regulated learning profile affected online mathematics learning engagement through a higher perceived academic control. Compared with students with low self-regulation learning profile, students with medium and high self-regulation learning profile can better adapt to the online mathematics learning environment, effectively mobilize their own motivation, use effective learning strategies, timely reflect on their learning results and seek feedback, which can improve their perceived academic control. At the same time, the individual’s perceived academic control can directly improve the degree of learning engagement. Thus, higher perceived academic control in the medium and high self-regulated learning profile directly predicted higher engagement in online mathematics learning compared with students in the low self-regulated learning profile.

### Implications

Our study has theoretical and practical implications. In terms of theoretical significance, to our knowledge, this is the first time that a combination of variable-centered and individual-centered approaches has been applied in order to determine the self-regulated learning patterns of junior high school students during online mathematics learning, as well as to examine the effect of perceived academic control on the association between the self-regulated learning profiles and learning engagement from a person-centered approach. Then, our study supported and further extended the motivation and engagement wheel model: self-regulated learning as adaptive cognition positively predicted perceived academic control as adaptive cognition.

From a practical point of view, this study provided guidance for interventions to improve the online learning engagement of junior high school students. First, improving students’ self-regulated learning level is an effective way to increase online learning engagement. Schools should consider incorporating students’ self-regulated learning into their curriculum. By using classroom games and dynamic mathematics software (Wijaya et al., 2020, 2022a), teachers can let students develop a better understanding of self-regulation strategies and stimulate their self-regulation learning. Mathematics teachers should provide specialized instruction and related training activities tailored to students with different self-regulated learning profiles. Second, protecting and enhancing students’ perceived academic control is another effective measure to increase students’ engagement in online learning. Schools should create an effective and appealing teaching platform for students (Wijaya and Weinhandl, 2022), which can provide individuals with a stable online learning environment and effectively affect students’ academic control. It is important for mathematics teachers to guide students in developing a positive attribution style, setting course objectives at a level that is appropriate for students’ cognitive development and needs, and improving their ability to predict and influence academic success. Doing so can increase students’ level of perceived academic control.

### Limitations and future directions

Despite the theoretical and practical implications, the limitations of our work should be recognized. First, the findings of our research may not be generalizable to all students, as the sample only consisted of those in the first and second grades of junior high school students. Future research can explore the potential types of self-regulated learning profiles for high school or college students, as well as the relationship between self-regulation and online learning engagement. Second, our study was based on a Chinese sample, which may differ in terms of self-regulated learning, compared to other educational contexts. Therefore, it is necessary to compare students’ self-regulated learning across different educational contexts to determine whether our findings are applicable. Third, the research method used in this study is a cross-sectional design, which cannot reveal changes in variables over time. A cross-lagged design can be used in future studies to investigate causal relationships between variables and analyze mediating effects between longitudinal data.

## Conclusion

Focusing on the learning context of online mathematics learning, the study found: (1) there were three potential types of self-regulated learning in junior high school students: high self-regulated learning, medium self-regulated learning, and low self-regulated learning; (2) Simple mediation analysis showed that perceived academic control partially mediated the relationship between self-regulated learning and online learning engagement; and (3) Multi-categorical mediation analysis showed that perceived academic control partially mediated the differences in the junior middle school students’ online learning engagement between the high/medium self-regulated learning and low self-regulated learning profile.

## Data availability statement

The raw data supporting the conclusions of this article will be made available by the authors, without undue reservation.

## Author contributions

WD: data analysis, manuscript draft, and revision work. ZL: study design, data collection, and manuscript draft. NJ: data interpretation and revision work. All authors contributed to the article and approved the submitted version.

## Funding

This work was supported by the major project of humanities and social sciences of Hebei Provincial Department of Education (No. ZD202109).

## Conflict of interest

The authors declare that the research was conducted in the absence of any commercial or financial relationships that could be construed as a potential conflict of interest.

## Publisher’s note

All claims expressed in this article are solely those of the authors and do not necessarily represent those of their affiliated organizations, or those of the publisher, the editors and the reviewers. Any product that may be evaluated in this article, or claim that may be made by its manufacturer, is not guaranteed or endorsed by the publisher.

## Footnotes

^1^MOE opens online learning platform to facilitate home study. http://en.moe.gov.cn/news/press_releases/202002/t20200201_417275.html
