# Supplementary material for: Self-regulated learning, online mathematics learning engagement, and perceived academic control among Chinese junior high school students during the COVID-19 pandemic: A latent profile analysis and mediation analysis
Source: Front Psychol. 2022 Nov 3;13:1042843. doi: 10.3389/fpsyg.2022.1042843 (PMC9670138; doi:10.3389/fpsyg.2022.1042843)
Supplement: Supplementary file 1 [file Table_1.DOCX]

Supplementary

TABLE1|Descriptive Statistics.

| Items | | Mean | Std.Dev. | SKewness | Kurtosis |
| --- | --- | --- | --- | --- | --- |
|  |  | Statistic | Statistic | Statistic | Statistic |
| Self-Regulated Learning | SRL1 | 3.750 | 1.008 | -0.598 | -0.045 |
|  | SRL2 | 3.750 | 1.029 | -0.663 | 0.054 |
|  | SRL3 | 3.689 | 1.165 | -0.651 | -0.406 |
|  | SRL4 | 4.136 | 1.024 | -1.206 | 1.061 |
|  | SRL5 | 4.404 | 0.886 | -1.515 | 1.919 |
|  | SRL6 | 4.166 | 1.062 | -1.208 | 0.668 |
|  | SRL7 | 4.229 | 0.989 | -1.392 | 1.696 |
|  | SRL8 | 3.946 | 1.101 | -0.972 | 0.325 |
|  | SRL9 | 3.963 | 1.124 | -1.08 | 0.56 |
|  | SRL10 | 3.175 | 1.284 | -0.277 | -0.971 |
|  | SRL11 | 3.617 | 1.208 | -0.584 | -0.509 |
|  | SRL12 | 3.437 | 1.259 | -0.458 | -0.78 |
|  | SRL13 | 2.900 | 1.326 | -0.014 | -1.07 |
|  | SRL14 | 4.605 | 0.712 | -2.04 | 4.282 |
|  | SRL15 | 4.189 | 0.975 | -1.271 | 1.315 |
|  | SRL16 | 3.780 | 1.149 | -0.69 | -0.279 |
|  | SRL17 | 3.780 | 1.14 | -0.742 | -0.219 |
|  | SRL18 | 3.771 | 1.122 | -0.689 | -0.253 |
|  | SRL19 | 3.944 | 1.036 | -0.889 | 0.258 |
|  | SRL20 | 3.678 | 1.053 | -0.572 | -0.085 |
|  | SRL21 | 3.446 | 1.23 | -0.414 | -0.779 |
|  | SRL22 | 3.586 | 1.201 | -0.629 | -0.364 |
| Online Mathematics Learning Engagement | OMLE1 | 4.021 | 1.121 | -0.953 | 0.002 |
|  | OMLE2 | 3.582 | 1.398 | -0.606 | -0.88 |
|  | OMLE3 | 4.206 | 0.963 | -1.291 | 1.244 |
|  | OMLE4 | 3.776 | 1.078 | -0.636 | -0.36 |
|  | OMLE5 | 3.914 | 1.254 | -1.024 | 0.03 |
|  | OMLE6 | 4.079 | 0.907 | -0.839 | 0.323 |
|  | OMLE7 | 2.692 | 1.289 | 0.234 | -1.076 |
|  | OMLE8 | 4.033 | 1.114 | -1.097 | 0.424 |
|  | OMLE9 | 3.706 | 1.123 | -0.715 | -0.085 |
|  | OMLE10 | 3.659 | 1.127 | -0.533 | -0.45 |
|  | OMLE11 | 3.897 | 1.273 | -0.834 | -0.507 |
|  | OMLE12 | 4.238 | 0.991 | -1.506 | 2.069 |
|  | OMLE13 | 4.598 | 0.706 | -2.016 | 4.545 |
| Perceived Academic Control | PAC1 | 3.722 | 1.091 | -0.715 | 0.095 |
|  | PAC2 | 4.194 | 0.983 | -1.228 | 1.125 |
|  | PAC3 | 2.100 | 1.166 | 0.801 | -0.251 |
|  | PAC4 | 4.362 | 0.878 | -1.485 | 2.043 |
|  | PAC5 | 1.680 | 0.964 | 1.496 | 1.884 |
|  | PAC6 | 1.862 | 1.098 | 1.139 | 0.412 |
|  | PAC7 | 3.853 | 1.118 | -0.909 | 0.152 |
|  | PAC8 | 2.222 | 1.217 | 0.689 | -0.565 |
